# SAHCS Guideline for the prevention, diagnosis and management of cryptococcal disease among persons living with HIV: Update to induction treatment for cryptococcal meningitis

**DOI:** 10.4102/sajhivmed.v27i1.1789

**Published:** 2026-03-18

**Authors:** Nelesh P. Govender, Graeme Meintjes, Jonathan Falconer, Kyla Murphy, Jeremy Nel, Helena Rabie, Lisa Frigati, Denasha L. Reddy, Halima Dawood, Ebrahim Variava, Samantha Potgieter, Tom Boyles, Sarah L. Stacey, Petho Mangena, Matamela C. Madua, Camilla Wattrus, Mahomed-Yunus S. Moosa

**Affiliations:** 1 Wits Mycology Division, School of Pathology, Faculty of Health Sciences, University of the Witwatersrand, Johannesburg, South Africa; 2 Institute for Infection and Immunity, City St. George’s University of London, London, United Kingdom; 3 MRC Centre for Medical Mycology, University of Exeter, Exeter, United Kingdom; 4 Blizard Institute, Queen Mary University of London, London, United Kingdom; 5 Department of Medicine and Institute of Infectious Disease and Molecular Medicine, Faculty of Health Sciences, University of Cape Town, Cape Town, South Africa; 6 Division of Infectious Diseases, Department of Medicine, School of Clinical Medicine, University of the Witwatersrand, Johannesburg, South Africa; 7 Division of Clinical Microbiology, Department of Medicine, School of Clinical Medicine, University of the Witwatersrand, Johannesburg, South Africa; 8 Department of Medicine, University of Cape Town, Cape Town, South Africa; 9 Neuroscience Institute, University of Cape Town, Cape Town, South Africa; 10 Department of Paediatrics, Stellenbosch University, Stellenbosch, South Africa; 11 Department of Paediatrics, Tygerberg Hospital, Cape Town, South Africa; 12 Vaccines and Infectious Diseases Analytics Research Unit (Wits-Vida), University of the Witwatersrand, Johannesburg, South Africa; 13 Infectious Disease Unit, Greys Hospital, Pietermaritzburg, South Africa; 14 Centre for the AIDS Programme of Research in South Africa, (CAPRISA), University of KwaZulu-Natal, Durban, South Africa; 15 Department of Internal Medicine, Faculty of Health Sciences, University of the Witwatersrand, Johannesburg, South Africa; 16 Klerksdorp Tshepong Hospital Complex, Klerksdorp, South Africa; 17 Perinatal HIV Research Unit, University of the Witwatersrand, Johannesburg, South Africa; 18 Department of Internal Medicine, Faculty of Health Sciences, University of the Free State, Bloemfontein, South Africa; 19 Clinical HIV Research Unit, Faculty of Health Sciences, University of the Witwatersrand, Johannesburg, South Africa; 20 Department of Medicine, Charlotte Maxeke Johannesburg Academic Hospital, Johannesburg, South Africa; 21 Department of Medicine, Polokwane Hospital, Polokwane, South Africa; 22 Department of Cardiology, Charlotte Maxeke Johannesburg Academic Hospital, Johannesburg, South Africa; 23 Southern African HIV Clinicians Society (SAHCS), Johannesburg, South Africa; 24 Department of Infectious Disease, Division of Internal Medicine, Nelson R Mandela School of Medicine, University of KwaZulu-Natal, Durban, South Africa

## Induction treatment recommendations

The Southern African HIV Clinicians Society (SAHCS) now recommends a single dose of liposomal amphotericin B (LAmB: 10 mg/kg) with 14 days of flucytosine (100 mg/kg/day in four divided doses) and fluconazole (1200 mg/day) as the first-line induction therapy for cryptococcal meningitis (see Table 1 for dosing). Aligned with the WHO 2022 guideline^1^ and the South African Standard Treatment Guidelines (2024),^2^ this is recommended as preferred induction treatment. This recommendation is based on the multicentre AMBisone Therapy Induction OptimisatioN-cryptococcal meningitis (AMBITION-cm) randomised controlled trial (*N* = 844), which demonstrated that a single high-dose LAmB-containing regimen was non-inferior to a regimen of 7 days of amphotericin B deoxycholate and flucytosine followed by fluconazole (10-week all-cause mortality: 24.8% vs 28.7%), with significantly fewer grade 3–4 adverse events, including fewer cases of anaemia, nephrotoxicity, and thrombophlebitis.^3^ The high-dose LAmB regimen was well tolerated, preferred by patients and providers, and cost-neutral overall because of reduced monitoring and supportive care needs. Amphotericin B deoxycholate plus flucytosine for 7 days, followed by 7 days of fluconazole, is the recommended alternative regimen if LAmB is unavailable. Amphotericin B deoxycholate plus fluconazole for 14 days is recommended if flucytosine is unavailable.

**TABLE 1:** Induction therapy doses of flucytosine, fluconazole, and amphotericin B, adjusted according to estimated glomerular filtration rates.

Antifungal agent	eGFR > 50 mL/min	eGFR 10–50 mL/min	eGFR < 10 mL/min	Haemodialysis
**Liposomal amphotericin B** †	10 mg/kg single dose	10 mg/kg single dose	10 mg/kg single dose	10 mg/kg single dose (not dialysed)
**Amphotericin B deoxycholate** †	1 mg/kg	1 mg/kg on alternate days‡	1 mg/kg on alternate days‡	1 mg/kg (can administer during dialysis)
**Fluconazole** (Adults and adolescents > 13 years and > 40 kg)	1200 mg daily	600 mg daily	600 mg daily	600 mg daily; dose after dialysis
**Fluconazole** (Children and adolescents < 13 years and < 40 kg)†	Stat dose of 25 mg/kg then 12 mg/kg/dose; max dose800 mg	6 mg/kg/dose; max dose200 mg	3 mg/kg/dose – 6 mg/kg/dose; max dose 200 mg	3 mg/kg/dose – 6 mg/kg/dose; max dose 00 mg
**Flucytosine?** (see Table 4 for specific dosing guidance)	25 mg/kg 6 hourly	25 mg/kg 12 hourly	25 mg/kg daily	25 mg/kg daily; dose after dialysis

*Source*: Adapted from Gilbert DN, Eliopoulos GM, Chambers HF, et al., editors. Sanford guide to antimicrobial therapy 2024. 54th ed. Sperryville, VA: Antimicrobial Therapy, Inc.; 2024.^5^

eGFR, estimated glomerular filtration rate.

‡ , Children with renal dysfunction should be discussed with an experienced clinician.

‡ , Amphotericin B deoxycholate is nephrotoxic but not renally excreted, so increased spacing is to reduce nephrotoxicity rather than to adjust for reduced renal clearance. If amphotericin B deoxycholate was intended to be given for 2 weeks, for example, alternate-day spacing over the 2-week period would mean that only 7 doses would be given in total.

There are no randomised controlled trials on cryptococcal meningitis in children, but there is nothing to suggest that outcomes with the AMBITION-cm regimen would differ from adults; therefore the same strategy is recommended. Although high-dose fluconazole has not been specifically studied in children or young adolescents, given the low weight of adults in the trial (median 53 kg, interquartile range [IQR]: 47–60), adolescents aged ≥ 13 years and weighing > 40 kg are recommended to receive adult dosing. There is no child-friendly formulation of flucytosine, but 500 mg tablets can be used in children weighing ≥ 17 kg. For children weighing < 17 kg, a single 500 mg tablet can be crushed in 50 mL of distilled water (or Ora-sweet, Ora-plus) to make a 10 mg/mL suspension, with the actual dose to be given calculated per kilogram body weight.

## Key practice points for the preferred regimen

The 2019 SAHCS Guideline for the prevention, diagnosis and management of cryptococcal disease among persons living with HIV^4^ should be used as a detailed reference for the administration, and toxicity prevention/management, of amphotericin B deoxycholate, fluconazole, and flucytosine. This update provides information on administration, and toxicity prevention and management for LAmB.

Key practice point

Liposomal amphotericin B is significantly different from amphotericin B deoxycholate. The daily doses are different, as are the reconstitution/ administration requirements and toxicity profiles. This should be highlighted to all clinicians, nurses, and pharmacists involved in its use.

**FIGURE 1:**
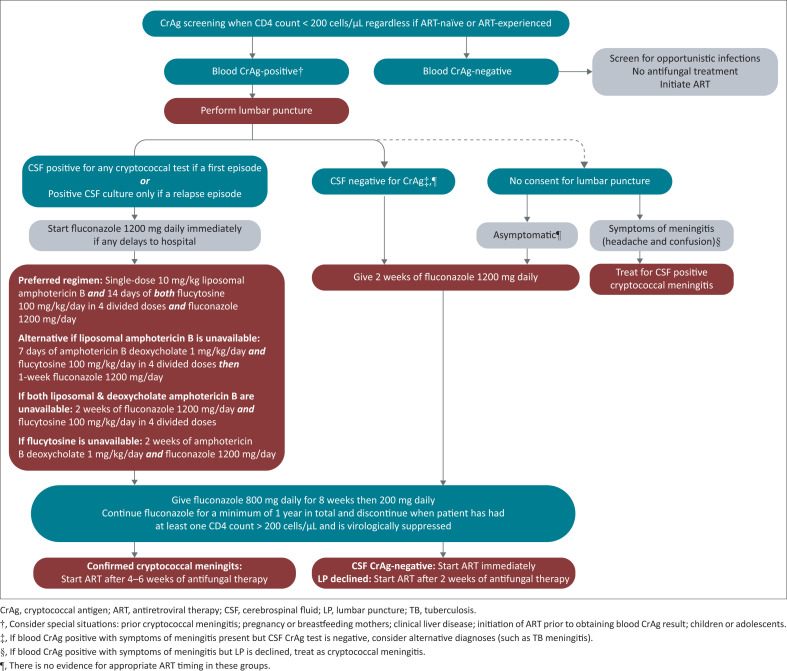
Screening and treatment algorithm for cryptococcal disease.

It is important to note that the use of single high-dose LAmB is dependent on the availability of both flucytosine and fluconazole to complete induction treatment. In the absence of flucytosine, it is necessary to revert to a 14-day course of amphotericin B deoxycholate and fluconazole, as detailed in the treatment algorithm (Figure 1). The availability of all three antifungals should be confirmed with the pharmacy prior to prescription.

### Administration of LAmB

The total dose of LAmB is calculated as a single dose of 10 mg/kg. This dose does not need to be adjusted in patients with a reduced estimated glomerular filtration rate (Table 1). LAmB powder (50 mg vials) should be stored below 25°C and protected from light during storage (Table 2). Each 50 mg vial should be aseptically reconstituted with 12 mL of sterile water for injection to yield a concentration of 4 mg/mL. The required volume of reconstituted LAmB should then be further diluted in 5% dextrose to a final concentration of 1–2 mg/mL. The reconstituted drug should be injected into 5% dextrose via the provided filters.

**TABLE 2:** Summary of administration and toxicity prevention and/or monitoring and/or management for amphotericin B (the full table for other induction agents are detailed in SAHCS 2019 guidelines).^4^

Scenario	Sub-recommendations
Key practice point: LAmB and amphotericin B deoxycholate are very different formulations and must not be confused.	
Administration of LAmB	Reconstitute each vial with 12 mL of sterile water to obtain a concentration of 4 mg/mL (assumes a 0.5 mL displacement volume for the powder). Shake the vial for 30 s to ensure the powder has dissolved. **For adults and adolecents > 13 years and > 40 kg:** LAmB should be reconstituted in sterile water; inject the calculated volume of reconstituted antifungal in water through the provided filters into 1 L of 5% dextrose and infuse over 2 h. The equivalent amount of 5% dextrose fluid will need to be removed prior to addition of the reconstituted antifungal volume. Administer within 24 h of reconstitution and ensure it is stored at 2°C – 8°C. The reconsituted bag does not need to be protected from light. Flush line with 5% dextrose before and after infusion. LAmB can be administered via a peripheral intravenous (IV) line if the concentration is < 2 mg/mL. A test dose is unnecessary. **For children and adolescents < 13 years and < 40 kg:** See Table 4 for dilution. Note: Follow Table 4 if 200 mL 5% dextrose is available. Follow Table 5 (Appendix 1) if 200 mL 5% dextrose is unavailable, and instead use 1 L 5% dextrose and 150 mL buretrol.
Administration of amphotericin B deoxycholate (adults and adolescents > 40 kg)	Amphotericin B deoxycholate powder should be reconstituted in sterile water; inject the calculated volume of reconstituted antifungal in water into 1 L of 5% dextrose water and administer within 24 h. Amphotericin B deoxycholate can be administered via a peripheral IV line if the solution contains ≤ 0.1 mg of amphotericin B deoxycholate per 1 mL of 5% dextrose water. A test dose is unnecessary. The solution should be infused over at least 4 h.
Administration of amphotericin B deoxycholate (children and adolescents < 40 kg)	Reconstitute each 50 mg vial with 10 mL sterile water to make a 5 mg/mL solution. Calculate the patient requirement in mL of reconstituted amphotericin B deoxycholate, that is, mL of reconstituted deoxycholate required = patient dose/5 Dilute with 5% dextrose; infusion should be over at least 4 h. Flush line with 5% dextrose before and after infusion.
Administration of flucytosine‡	Flucytosine comes in 500 mg tablets (or capsules, depending on the manufacturer). These should be administered by weight-based dosing. Suggested dosing regimens are shown in Table 3 and Table 4. If a patient is unable to swallow, tablets can be crushed and administered via a nasogastric tube. In a child weighing < 17 kg, a single 500 mg tablet can be mixed with 50 mL of Ora-sweet, Ora-plus, or distilled water, and delivered as a 10 mg/mL solution, as per the dosing schedule in Table 4.
Prevention of amphotericin B-related toxicities (both formulations)	Adults should be pre-hydrated with 1 L of normal saline containing one ampoule of potassium chloride (20 mmol) infused over 2 h before the amphotericin B infusion§ Twice daily oral potassium and daily oral magnesium supplementation should be administered to adults until 2 days after the final dose of amphotericin B. To minimise the risk of phlebitis, lines should be flushed with 5% dextrose immediately after the amphotericin B infusion is complete and the infusion bag should not be left attached to the intravenous administration set after the infusion is complete.
Monitoring of patients receiving liposomal amphotericin B and flucytosine	**Days 0 and 3:** creatinine and potassium (and magnesium, if available). **Days 0 and 7:** full blood count (with a differential count if available). **Day 3:** full blood count and differential count can be considered when flucytosine is used, especially if baseline abnormalities exist. Flucytosine may cause bone marrow suppression but this is uncommon with a short duration of use, the current suggested dosing schedule and in the absence of renal impairment. Fluid input and output monitoring.
Management of amphotericin B-related toxicities (both formulations)	**Infusion reactions:** Rigours and febrile reactions are common. They can be treated by slowing the rate of infusion and administering paracetamol 1 g 30 min before the infusion (if severe, hydrocortisone 25 mg IV can be given before subsequent infusions). **Renal impairment:** As LAmB is administered as a single dose, no subsequent dose adjustment is necessary. Otherwise close monitoring and supportive treatment is normally sufficient. For recommendations regarding amphotericin B deoxycholate, refer to recommendation 4 (renal impairment section) of the 2019 guidelines.^4^ **Anaemia:** Transfuse according to local guidelines. **Potassium and magnesium impairment:** For significant hypokalaemia (serum K+ < 3.3 mmol/L), additional intravenous replacement is required: up to two ampoules of potassium chloride (20 mmol K+ per 10 mL ampoule) in 1 L of normal saline 8 hourly. Among those who develop hypokalaemia, serum potassium should be monitored daily until it is resolved. If hypokalaemia remains uncorrected, serum magnesium should be checked (if this test is available) and/or oral magnesium supplementation should be doubled. Intravenous magnesium sulphate may be considered for persistent hypokalaemia and hypomagnesaemia.

LAmB, liposomal amphotericin B.

‡ , For adolescents and children, doses should be calculated by body weight.

§ , For children and adolescents, normal saline, with one ampoule of potassium chloride (20 mmol) added per 1 L of fluid, should be infused at 10 mL/kg – 15 mL/kg over 2–4 h (not more than 1 L) prior to amphotericin B administration. If saline is unavailable, then other parenteral rehydration solutions, for example Ringer’s lactate, that already contain potassium can be used.

LAmB should ***never*** be mixed with saline-containing solutions as this will cause precipitation. The prepared infusion should be administered over 2 h via a dedicated intravenous line. If an existing intravenous line is used, it must be flushed with 5% dextrose prior to infusion of LAmB. A test dose is not required, and protection from light during infusion is not necessary. Once prepared, the reconstituted LAmB solution should be used promptly or stored at 2°C – 8°C and infused within 24 h. After completion of the infusion, the line should be flushed with 5% dextrose solution before further use. These recommendations are summarised in Table 2. Simplified dosing and reconstitution guidance is provided in Table 3 (adults) and Table 4 (children and adolescents).

**TABLE 3:** Simplified dosing and reconstitution guidance for LAmB and flucytosine.

Liposomal amphotericin B (10 mg/kg) IV (in 5% dextrose)	Patient weight (kg)	Dose (mg)	Number of 50 mg vials	Volume (to remove from IV bag before adding LAmB)	Flucytosine (100 mg/kg/day in four divided doses) Oral	Patient weight (kg)	Total daily dose (mg)	Number of 500 mg pills/day	Suggested dosing schedule
	41–45	450	9	112.5 mL		40–44	4000	8	2–2–2–2
	46–50	500	10	125 mL		45–49	4500	9	3–2–2–2
Scan QR code to learn how to reconstitute LAmB 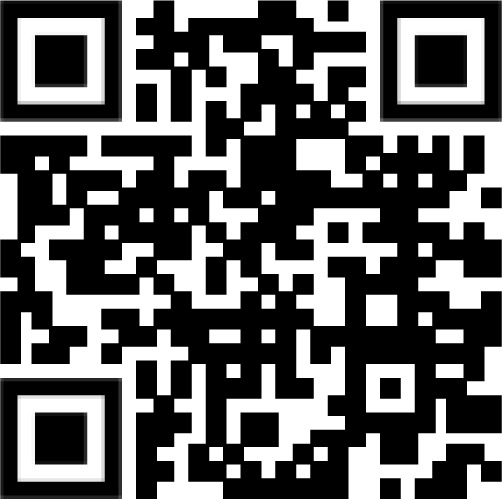	51–55	550	11	137.5 mL	Scan QR code to learn how to manage renal and blood toxicity 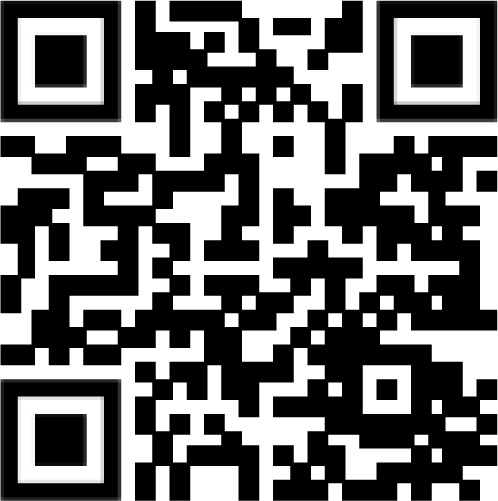	50–54	5000	10	3–2–3–2
	56–60	600	12	150 mL		55–59	5500	11	3–3–3–2
	61–65	650	13	162.5 mL		60–64	6000	12	3–3–3–3
	66–70	700	14	175 mL		65–69	6500	13	4–3–3–3
	71–75	750	15	187.5 mL		70–74	7000	14	4–3–4–3
	76–80	800	16	200 mL		75–79	7500	15	4–4–4–3
	81–85	850	17	212.5 mL		80–84	8000	16	4–4–4–4
	86–90	900	18	225 mL		85–89	8500	17	5–4–4–4
	91–95	950	19	237.5 mL		90–94	9000	18	5–4–5–4
Administer in 1 L of 5% dextrose fluid over 2 h For weights > 95 kg, calculate dose: 10 mg x weight (kg), then ***round up*** to next 50 mg					For weights ≥ 95 kg, calculate dose: 100 mg × weight (kg), then ***round down*** to next 500 mg/day				

*Source:* Adapted from the SHARE-CM/IMPRINT project teaching material. Available from https://www.differentiatedservicedelivery.org/resources/the-global-advanced-hiv-disease-toolkit/^6^

IV, intravenous.

**TABLE 4:** Simplified dosing of flucytosine, and simplified dosing and infusion of LAmB using a 200 mL bag of 5% dextrose for children and adolescents < 40 kg.

Flucytosine					Liposomal amphotericin B (LAmB)								
Patient weight (kg)	Total daily dose (mg)	Number of 500 mg pills needed per day	Set of 500 mg/tablet flucytosine pills taken per mouth	Millilitres of 10 mg/mL solution taken four times a day	(A) Weight (kg)	(B) Number vials of to reconstitute	(C) Volume of 5% dextrose to be removed from 200 mL bag	(D) mL of reconstituted LAmB	(E) Total volume to be infused	(F) Infusion	(G) Infusion rate	Dose in mg	Patient weight (kg)
5–6	600	-	-	15 mL	5–6	2	116	16	100 mL	4	25 mL/h	62.5	5–6
7–8	800	-	-	20 mL	7–8	2	122	22	100 mL	4	25 mL /h	87.5	7–8
9–10	1000	-	-	25 mL	9–10	2	125	25	100 mL	2	20 mL /h	100	9–10
11–12	1200	-	-	30 mL	11–12	3	132	32	100 mL	2	20 mL /h	125	11–12
13–14	1400	-	-	35 mL	13–14	3	137.5	37.5	100 mL	2	20 mL /h	150	13–14
15–16	1600	-	-	40 mL	15–16	3	137.5	37.5	100 mL	2	20 mL /h	150	15–16
17–20	2000	4	1–1–1–1	-	17–20	4	50	50	200 mL	4	50 mL /h	200	17–20
21–26	2500	5	2–1–1–1	-	21–26	5	62.5	62.5	200 mL	4	50 mL /h	250	21–26
27–30	3000	6	2–1–2–1	-	27–30	6	75	75	200 mL	2	100 mL /h	300	27–30
31–32	3500	7	2–2–2–1	-	31–32	6	75	75	200 mL	2	100 mL /h	300	31–32
33–36	3500	7	2–2–2–1	-	33–36	7	87.5	87.5	200 mL	2	100 mL /h	350	33–36
37–40	4000	8	2–2–2–2	-	37–40	8	100	100	200 mL	2	100 mL /h	400	37–40
Where possible suspensions should be made up by a pharmacist. Make a 10 mg/mL solution by adding 2 × 500 mg in 100 mL of sterile water (10 mg/mL). The solution should be refrigerated, protected from light and not be kept for more than 7 days.					Find the appropriate weight band **(A)** Identify the number of vials **(B)** needed and reconstitute each vail with 12 mL of sterile water Take a 200 mL bag of 5% dextrose Remove the appropriate volume of 5% dextrose from the 5% dextrose bag **(C)** Add the volume of LAmB needed **(D)** to the 200 mL bag of 5% dextrose and discard excess LAmB, where appropriate. The total volume to be infused is **(E)** and the infusion time **(F)** and set infusion rate **(G)**								

LAmB, liposomal amphotericin B.

### LAmB toxicity prevention and management

LAmB is associated with significantly less nephrotoxicity, and fewer electrolyte disturbances and infusion-related reactions compared to amphotericin B deoxycholate; however, monitoring and supportive care remain important. Pre-hydration is recommended with 1 L of normal saline containing one ampoule of potassium chloride (20 mmol potassium [K+] per 10 mL ampoule) over 2 h prior to infusion to reduce the risk of kidney injury and hypokalaemia. The volume of pre-hydration needed in children and adolescents aged < 13 years, and weighing < 40 kg is 10 mL/kg – 15 mL/kg, with a maximum volume of 1 L over 2–4 h. The child’s weight and nutrition status should be considered when deciding on the infusion rate.

Pre-emptive potassium and magnesium oral supplementation is advised for the first 3 days, unless baseline testing identifies hyperkalaemia (K+ > 5.5 mmol/L). Patients should be given 1200 mg of potassium chloride orally twice daily (equivalent to 16 mmol oral potassium and up to 1500 mg magnesium chloride orally daily if available.

In children, the oral potassium chloride dose should be guided by serum potassium levels, and renal function needs to be considered. If the renal function and the serum potassium level are normal, 1 mEq/kg/day – 2 mEq/kg/day (75 mg/kg/day – 150 mg/kg/day) of potassium divided into 2–3 doses can be considered for the first 3 days and carefully monitored. The dose of magnesium trace element mix for children under 10 kg is 2.5 mL per day, and 5 mL per day if over 10 kg. If magnesium trace element mix is unavailable, the intravenous preparation of magnesium sulphate 50% can be used ***orally*** at 0.2 mL/kg as a once daily dose.

Serum potassium, magnesium and creatinine levels should be monitored at baseline, as well as 3 days after treatment. Infusion-related reactions (e.g. fever, chills, rigours) may occur; these are not allergic reactions, and they can be managed by temporarily slowing the infusion rate, administering paracetamol, or using antihistamines if needed. Phlebitis is rare, but can be minimised by using a dedicated intravenous line, flushing the line with 5% dextrose after the infusion, and removing or re-siting the line if there is redness or discomfort.
